# Identification of a Novel Serum Proteomic Signature for Primary Sjögren’s Syndrome

**DOI:** 10.3389/fimmu.2021.631539

**Published:** 2021-02-23

**Authors:** Guillaume Padern, Claire Duflos, Rosanna Ferreira, Said Assou, Philippe Guilpain, Alexandre Thibault Jacques Maria, Radjiv Goulabchand, Pascale Galea, Maja Jurtela, Christian Jorgensen, Yves-Marie Pers

**Affiliations:** ^1^ IRMB, University of Montpellier, INSERM, CHU Montpellier, Montpellier, France; ^2^ Clinical Research and Epidemiology Unit, CHU Montpellier, Montpellier University, Montpellier, France; ^3^ Internal Medicine and Multi-Organic Diseases Department, Hôpital Saint Éloi, CHU Montpellier, Montpellier, France; ^4^ Internal Medicine Department, Caremeau University Hospital, Nîmes, France; ^5^ BioRad Laboratory, Research and Development Department, Montpellier, France

**Keywords:** primary Sjögren syndrome, systemic lupus erythematosus, rheumatoid arthritis, biomarkers, proteomics

## Abstract

**Context:**

Primary Sjögren’s syndrome (pSS) is a complex heterogeneous autoimmune disease (AID) which can mimic rheumatoid arthritis (RA) or systemic lupus erythematosus (SLE). Our exploratory study investigated serum biomarkers that may discriminate pSS from RA and SLE.

**Methods:**

Serum concentrations of 63 biomarkers involved in immune cell trafficking, inflammatory response, cellular movement, and cell-to-cell signaling were measured in AID patients, included prospectively into the study at the Montpellier University Hospital. A multivariate analysis by multiple logistic regression was performed, and discriminative power assessed using logistic regression adjusted on significant demographic factors.

**Results:**

Among the 95 patients enrolled, 42 suffered from pSS, 28 from RA, and 25 from SLE. Statistical analysis showed that concentrations of BDNF (OR = 0.493 with 95% CI [0.273–0.891]; p = 0.0193) and I-TAC/CXCL11 (OR = 1.344 with 95% CI [1.027–1.76]; p = 0.0314) can significantly discriminate pSS from RA. Similarly, greater concentrations of sCD163 (OR = 0.803 with 95% CI [0.649–0.994]; p = 0.0436), Fractalkine/CX3CL1 (OR = 0.534 with 95% CI [0.287–0. 991]; p = 0.0466), MCP-1/CCL2 (OR = 0.839 with 95% CI [0.732–0.962]; p = 0.0121), and TNFa (OR = 0.479 with 95% CI [0.247–0.928]; p = 0.0292) were associated with SLE diagnosis compared to pSS. In addition, the combination of low concentrations of BDNF and Fractalkine/CX3CL1 was highly specific for pSS (specificity 96.2%; positive predictive value 80%) compared to RA and SLE, as well as the combination of high concentrations of I-TAC/CXCL11 and low concentrations of sCD163 (specificity 98.1%; positive predictive value 75%).

**Conclusion:**

Our study highlights biomarkers potentially involved in pSS, RA, and SLE pathophysiology that could be useful for developing a pSS-specific diagnostic tool.

## Introduction

Primary Sjögren’s syndrome (pSS) is a complex heterogeneous autoimmune disease (AID) characterized by salivary and/or ocular dryness (sicca syndrome) related to lymphoid infiltration of exocrine glands. pSS is distinguished from secondary Sjögren’s syndrome (sSS), which often coexists with systemic lupus erythematosus (SLE) and rheumatoid arthritis (RA) (1). While pSS, RA, and SLE have distinct clinical and pathophysiological features (2–4), patients may initially present with sicca symptoms, and over the course of the disease, the specific underlying condition will then manifest (5, 6). Accurate diagnosis is often challenging in patients with overlapping disease entities. In addition, the lack of specificity of lymphocytic sialadenitis and anti-SSa/b antibodies (7, 8) can complicate pSS diagnosis. Glandular epithelial cells play a central pathophysiological role in the development of autoimmune epithelitis, especially concerning antigen presentation of Ro/SSA- and La/SSB-protein complexes. Both the innate and the adaptive immune system are involved in the disease initiation and maintenance of the immune response. An “interferon signature” is observed in the salivary glands and T-cell derived cytokines (Th1/Th2 polarization, Th17, and regulatory T cells) play a central role in the pathophysiology of pSS. Moreover, through activation of various CD4+ T-helper cell subsets, B cells are involved in auto-antibody production and the formation of ectopic germinal center-like structures associated with malignant transformation to Non-Hodgkin lymphoma (9).

Identifying new biomarkers involved in these AIDs and developing pSS diagnosis or prognosis methods is urgently needed (10). Single biomarker approaches are overwhelmed by their limitations and the complexity of AID physiopathology. Proteomic techniques consider the multifactorial processes involved, and their more global approach may provide a more complete picture of the disease (11). Multiplex immunoassays are promising and have allowed for the identification of interesting biomarkers in AIDs (12–15). Such an approach may soon be available in clinical practice despite limitations such as interference by highly concentrated proteins and cross-reactivity (16).

Several recently launched research initiatives are attempting to identify clinical and immunological signatures in pSS patients (17). Furthermore, many potential biomarkers of pSS have emerged for several years (18–22), but none of them have been validated yet (23). In comparisons with RA and SLE, a better characterization of the pathophysiological mechanisms involved in pSS seems useful to better discriminate these AIDs. Therefore, we conducted a proteomic study investigating the concentration of several serum biomarkers in pSS, RA, and SLE patients.

## Patients and Methods

### Study Population

This transversal study was conducted in the Rheumatology and Internal Medicine departments of Montpellier University Hospital. Patients were recruited consecutively and prospectively during 5 months between October 2016 and February 2017. They all met the international classification criteria for pSS (2), RA (3), or SLE (24). RA and SLE patients with sSS were excluded. Written informed consent was obtained from all patients, after which a blood sample was collected. The local ethics committee approved all procedures in accordance with international Helsinki regulations (Comité de Protection des Personnes Sud Méditerranée IV: DC-2015-2584).

### Data Collection

On the day of inclusion, the following data were obtained from each patient’s computerized medical record: age, gender, smoking status, date of diagnosis, articular and extra-articular autoimmune symptoms, disease severity, accessory salivary gland biopsy results (pSS only), autoantibody presence and specificity, cardiovascular risk factors (diabetes, hypertension, smoking, dyslipidemia, and chronic renal failure with a glomerular filtration rate below 30ml/min), bone erosions, C-reactive protein (CRP) levels, and ongoing specific AID therapy. At the time of inclusion, measurement of disease activity was performed using ESSDAI score (25) for pSS, DAS28-CRP score (26) for RA and SLEDAI score (27) for SLE. Low disease activity was defined by an ESSDAI score 5, a DAS28-CRP score 3.2, or a SLEDAI score ≤5. Moderate disease activity was defined by an ESSDAI score between 5 and 13 inclusive, a DAS28-CRP score 3.2 and 5.1, or a SLEDAI score between 6 and 10 inclusive. High disease activity was defined by an ESSDAI score 13, a DAS28-CRP score 5.1, or a SLEDAI score >10.

### Determination of Biomarkers Serum Concentration

Blood samples were stored for 30 min at room temperature (RT) before being centrifuged (2,000g, 10min, RT), frozen at −80°C, and stored in the biological resource center of Montpellier University Hospital (Pr Sylvain LEHMANN, NFS 96-900 and ISO 9001 standards, BB-0033-00031) until their use in the biomarkers assay.

Serum protein concentrations of 63 biomarkers were measured using Bio-Plex Pro™ Human Chemokine 40-plex Panel (Bio-Rad) for CCL1, CCL2 (Monocyte Chemoattractant Protein 1 or MCP-1), CCL3, CXCL5, CCL7, CCL8, CCL11 (Eotaxin), CCL13, CCL15, CCL17 (Thymus- and Activation-Regulated Chemokine or TARC), CCL19, CCL20, CCL21, CCL22, CCL23, CCL24, CCL25 (Eotaxin-2), CCL26 (Eotaxin-3), CCL27, CXCL1, CXCL2, CXCL6, CXCL9, CXCL10, CXCL11 (Inducible T-cell Alpha Chemoattractant or I-TAC), CXCL12, CXCL13, CXCL16, CX3CL1, Granulocyte-macrophage colony-stimulating factor (GM-CSF), IL1-beta, IL-2, IL-4, IL-6, IL-8, IL-10, IL-16, Macrophage migration Inhibitory Factor (MIF), Tumor Necrosis Factor Alpha (TNFa), and Interferon gamma (IFNg). Quality control was validated for 34 biomarkers, and data for the six non-validated biomarkers were rejected (CCL-26, IL1-beta, IL-2, IL-6, IL-10, and IFNg). The Bio-Plex Pro™ Human Inflammation Panel 1, 37-Plex (Bio-Rad) was validated for the dosage of A Proliferation-Inducing Ligand (APRIL), B Cell Activating Factor (BAFF), sCD30, sCD163, Chitinase 3-like 1, soluble IL-6 Receptor alpha (sIL-6Ra), soluble IL-6 Receptor beta (sIL-6Rb or gp130), Matrix Metalloproteinase-2 (MMP-2), Matrix Metalloproteinase-3 (MMP-3), Osteocalcin, Osteopontin (SPP1), Pentraxin-3, soluble TNF Receptor 1 (sTNF-R1), soluble TNF Receptor 2 (sTNF-R2), and TNF-related Weak inducer of apoptosis (TWEAK). Enzyme-linked immunosorbent assay (ELISA) kits were used for the remaining biomarkers: galectin binding protein 3 (LGalS3P) form Abnova, Fatty Acid-Binding Protein 4 (FABP4) from Biovendor, Hydroxyproline (HDP) from Cusabio, pre-Haptoglobin 2 (preHp2), from Bio-Rad (28), Oxidized low-density lipoprotein (OxLDL) from Mercodia, Secreted Phosphoprotein 1 (SPP1), Thrombospondin 2 (TPS2), adiponectin, hyaluronic acid (HA), Cathepsin S (CTSS), Brain Derived Neurotrophic Factor (BDNF), Secreted Protein Acidic Rich Cysteine (SPARC), haptoglobin, sCD14, and Mannose Binding Lectin 2 (MBL2) from R&D Systems. The osteopontin assay (SPP1) was performed using two different kits: an ELISA kit (R&D systems) whose result is reported under “SPP1”, and the Bio-Plex kit Pro™ Human Inflammation Panel 1, 37-Plex (BioRad) whose result is reported under “osteopontin” (**Table S1, Supplemental Data**). Reproducibility and sensitivity of all ELISA kits were first validated before performing serum biomarker assays with patient samples. Biomarker assays were performed in duplicate for each sample according to the recommendations of manufacturers.

**Figure 1:**
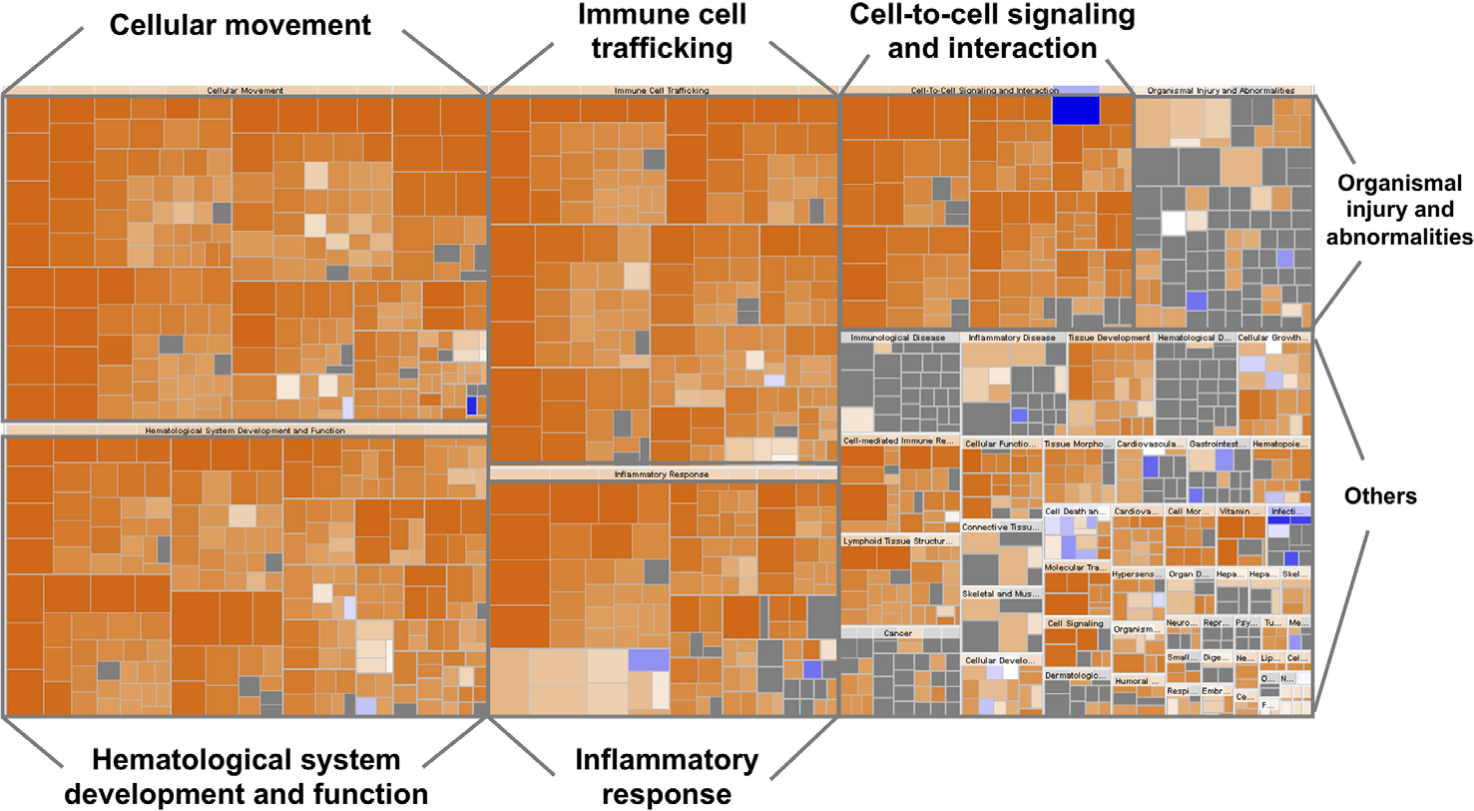
Heatmap representing the classification of diseases and functions by ingenuity pathway analysis (IPA). The visualization is a hierarchical heat map of functional categories generated by IPA software in which the major boxes represent a family (or category) of related functions. Within each box, each individual rectangle is a sub-function linked to the biological function of a group of proteins. The size of a rectangle is correlated with increasing overlap significance among the proteins members of the related function and the query proteins. The color scheme shown is based on z-scores, with activation in orange, inhibition in blue and undetermined functions in gray. Darker shades indicate higher absolute z-score heat map. For instance, most ‘Inflammatory response’, ‘Cellular movement’, ‘Immune cell trafficking’, ‘Cell-to-cell signaling and interaction’, ‘Hematological system development and function’ proteins were over-represented.

The top canonical pathways and the gene ontology analysis of the 63 biomarkers were functionally categorized using the ingenuity pathway analysis (IPA) software (QIAGEN Inc., https://www.qiagenbioinformatics.com/products/ingenuitypathway-analysis) (29). A large portion of this list is involved in hematological system development and function, immune cell trafficking, inflammatory response, cellular movement, and cell-to-cell signaling (**Figure 1**).

### Statistical Analysis

A simple descriptive analysis was performed on the entire study population and then by group. Groups were initially compared using univariate tests. Age and sex were considered clinically pertinent confounding factors and were included in our principal analysis if they were statistically significant in the univariate analysis.

The association between biomarkers and the risk of presenting with pSS rather than RA was assessed using separate logistic regressions for each biomarker. When the log-linearity hypothesis was met, to provide readable odds ratios (OR), biomarkers were specified in the model using the most convenient unit of concentration, ranging from 10 to 10,000 original units, depending on the biomarker. When the log-linearity hypothesis was not met, biomarkers were specified in the model as categorical variables. Thresholds were the quantiles determining six balanced groups. Finally, classes with comparable OR were grouped together.

The association between biomarkers and the risk of presenting with pSS rather than SLE was assessed using the same method. In addition, since the SLE patients were clinically and statistically younger than pSS patients, the logistic regressions were adjusted on age.

Samples were removed from the analysis if the concentration of a biomarker varied by more than 20% between technical duplicates (coefficient of variation > 20%) or if the concentration was outside the test detection range (**Table S2, Supplemental Data**). All statistical tests were two-tailed with a Type I error of 0.05. Analyses were performed using SAS^®^ Version 7.12 HF4 software.

## Results

### Study Population

Ninety-five patients were prospectively enrolled in the study between October 2016 and February 2017: 42 pSS patients, 28 RA patients, and 25 SLE patients. Population characteristics are summarized in **Table 1**, and biological diagnostic criteria in **Supplemental Table S3**. The median age of pSS patients (62.5 years) did not differ from those in the RA group (60.5 years), while both pSS and RA patients were significantly older than those in the SLE group (40 years, Q25: 31.5 – Q75: 50.5; p<0.001). Mean disease duration was significantly shorter in pSS patients (9.2 ± 8.6 years) compared to RA patients (15.8 ± 9.3 years; p<0.01) but did not differ in comparison with the SLE patients (12.1 ± 9.7 years; p = 0.53). Low disease activity was common in all three groups: 76.2% in pSS patients, 58.3% in RA patients, and 76% in SLE patients. As expected, more patients were treated by biologic drugs in the RA group compared to the pSS and SLE groups (p<0.001). Corticosteroid treatment did not differ among groups (p = 0.62). SLE patients were more often treated by synthetic Drug Modifying Anti-Rheumatic Diseases (DMARDs) than the others (p = 0.04). Likewise, SLE patients were treated by mycophenolate mofetil (MMF) or azathioprine (AZA) more than pSS and RA patients (for MMF p<0.001; for AZA p = 0.02). In contrast, the RA group included significantly more patients treated with methotrexate (p = 0.01) and fewer patients treated with hydroxychloroquine (p = 0.02) compared to the pSS and SLE groups.

**Table 1:** Characteristics of patients included in the study.

Characteristics	pSS	RA	SLE	p
Patients (n)	42	28	25	NA
Female gender (%)	40 (95.2%)	24 (85.7%)	24 (96%)	0.35
Median age (year; IQR)	62.5 (54.7;71.2)	60.5 (53; 73.5)	40 (31.5; 50.5)	< 0.01
Smoking patients (%)	4 (8.3%)	3 (10.3%)	5 (18.5%)	0.45
Mean BMI (kg/m2 ± SD)	27.6 ± 7.5	26.2 ± 5.7	25.4 ± 5.8	0.52
Cardiovascular risk factor				
No cardiovascular risk factor	22 (52.4%)	9 (32.1%)	11 (44%)	0.25
1 or 2 cardiovascular risk factors	17 (40.5%)	14 (50%)	12 (48%)	0.70
3 or 4 cardiovascular risk factors	3 (7.1%)	5 (17.9%)	2 (8%)	0.39
SSa and/or SSb positivity (%)	16 (38.1%)	0 (0%)	8 (32%)	< 0.01
Mean disease duration (year ± SD)	9.2 ± 8.6	15.8 ± 9.3	12.1 ± 9.7	0.01
Disease status				
Low disease activity	32 (76.2%)	14 (58.3%)	19 (76%)	0.25
Moderate disease activity	6 (14.3%)	8 (33.3%)	6 (24%)	0.26
High disease activity	4 (9.5%)	2 (8.3%)	0	0.36
Medications				
No treatment (%)	9 (27.3%)	2 (7.7%)	8 (32%)	0.08
Steroids (%)	8 (24.2%)	7 (26.9%)	4 (16%)	0.62
All synthetic DMARDs (%)	21 (63.6%)	15 (57.7%)	22 (88%)	0.04
Methotrexate (%)	6 (18.2%)	12 (46.2%)	3 (12%)	0.01
Sulfasalazine (%)	3 (9.1%)	1 (3.8%)	0	0.38
Leflunomide (%)	2 (6.1%)	1 (3.8%)	0	0.77
Hydroxychloroquine (%)	9 (27.3%)	1 (3.8%)	9 (36%)	0.02
Ciclosporin (%)	1 (3%)	0	0	> 0.99
Mycophenolate (%)	0	0	6 (24%)	0.01
Azathioprine (%)	0	0	3 (12%)	0.02
Tacrolimus (%)	0	0	1 (4%)	0.30
All biologic DMARDs (%)	3 (9.1%)	19 (73.1%)	1 (4%)	< 0.01
Rituximab (%)	3 (9.1%)	3 (11.5%)	1 (4%)	0.70
TNF inhibitors (%)	0	6 (23.1%)	0	0.01
Tocilizumab (%)	0	7 (27%)	0	0.01
Abatacept (%)	0	3 (11.5%)	0	0.05

Representation of the three autoimmune diseases groups: pSS, RA, and SLE. Comparisons between groups were done using either a Kruskall-Wallis test or a Chi-2 test (or a Fisher’s exact test when conditions necessary for Chi-2 were not met). Results from these tests are in column “p”.

pSS, primary Sjogren syndrome; RA, rheumatoid arthritis; SLE, systemic lupus erythematosus; IQR, interquartile range (Q1–Q3); DMARDs, Disease Modifying Anti-Rheumatic Drugs; NA, Not Applicable.

### Biomarkers Associated With pSS *Versus* RA

Identification of markers distinguishing pSS versus RA or SLE was determined by logistic regression analysis. The analysis was age-adjusted for the comparison with SLE patient samples. Results are presented as a function of the increase in each biomarker concentration. All results are available in **Table 2**.

**Table 2:** Likelihood of primary Sjogren syndrome (pSS) diagnosis versus rheumatoid arthritis (RA) or Systemic Lupus Erythematosus (SLE) as a function of an increase in biomarker concentration.

Biomarker name / Value of unit increase in serum concentration			pSS probability vs RA		pSS probability vs SLE	
			OR [95% CI]	p	**OR [95% CI]**	**p**
SPP1 / +10 ng/ml			1.067 [0.876–1.299]	0.5193	1.035 [0.787–1.36]	0.8076
HDP / +100 ng/ml			1.057 [0.974–1.147]	0.1835	0.9 [0.817–0.991]	0.0318
TPS2 / +10 ng/ml			1.059 [0.716–1.567]	0.7737	0.697 [0.433–1.122]	0.1371
preHp2 / +100 ng/ml			1.019 [0.877–1.183]	0.8066	0.859 [0.686–1.074]	0.1827
LGalS3P/ +1,000 ng/ml			1.116 [0.982–1.268]	0.092	1.029 [0.886–1.196]	0.7042
Adiponectin / +1,000 ng/ml			0.954 [0.87–1.047]	0.3191	0.958 [0.845–1.086]	0.5009
oxLDL/ +10,000 mU/ml			1.034 [0.822–1.301]	0.7737	1.093 [0.815–1.465]	0.5538
Hyaluronic Acid / +10 ng/ml			0.936 [0.863–1.015]	0.1109	1.137 [0.948–1.363]	0.1662
RA: FABP4 / +10 ng/ml		SLE: FABP4 Tertile 1	1.033 [0.9–1.186]	0.6408	0.32 [0.063–1.631]	0.1704
		SLE: FABP4 Tertile 2			0.459 [0.086–2.459]	0.3632
RA: CTSS / +1000pg/ml		SLE: CTSS Classes 0, 1, 2	0.939 [0.771–1.143]	0.5289	3.123 [0.563–17.314]	0.1925
		SLE: CTSS Classes 3, 4			14.064 [1.56–126.806]	0.0185
BDNF / +10,000pg/ml			0.493 [0.273–0.891]	0.0193	0.817 [0.406–1.647]	0.5728
RA: SPARC / +100ng/ml		SLE: SPARC Classes 0, 1, 2	1.065 [0.877–1.293]	0.5263	0.311 [0.031–3.173]	0.3246
		SLE: SPARC Class 3	1.794 [0.073–44.11]	0.7205		
		SLE: SPARC Class 4	0.035 [0.002–0.66]	0.0253		
Haptoglobin / +10,000 ng/ml			1.015 [1.002–1.029]	0.0262	1.004 [0.991–1.018]	0.5438
RA: sCD14 Tertile 1		SLE: sCD14+10,000 pg/ml	0.825 [0.245–2.783]	0.757	0.99 [0.975–1.005]	0.1907
RA: sCD14 Tertile 2			0.779 [0.243–2.501]	0.675		
MBL2 / +100 ng/ml			1.032 [0.976–1.09]	0.2679	1.055 [0.959–1.161]	0.2691
APRIL/TNFSF13 / +10,000 pg/ml			1.059 [0.952–1.177]	0.2921	0.995 [0.931–1.063]	0.8788
BAFF/TNFSF13B / +10,000 pg/ml			0.745 [0.53–1.047]	0.0898	0.704 [0.408–1.215]	0.2076
sCD30/TNFRSF8 / +100 pg/ml			1.045 [0.841–1.3]	0.6903	0.806 [0.631–1.03]	0.0848
sCD163 / +10,000 pg/ml			1.138 [0.96–1.35]	0.1364	0.803 [0.649–0.994]	0.0436
Chitinase 3 like / +1,000 pg/ml			0.972 [0.922–1.025]	0.3004	0.949 [0.881–1.023]	0.1699
gp130/sIL-6Rb / +1,000 pg/ml			1.031 [0.98–1.084]	0.2383	1.007 [0.948–1.069]	0.8271
sIL-6Ra / +1,000 pg/ml			0.901 [0.803–1.012]	0.0776	0.856 [0.698–1.049]	0.1329
MMP-2 / +1,000 pg/ml			1.025 [0.975–1.077]	0.3314	1.009 [0.947–1.075]	0.7793
MMP-3 / +1,000 pg/ml			1.007 [0.898–1.13]	0.9025	1.043 [0.899–1.209]	0.579
Osteocalcin / +100 pg/ml			0.96 [0.903–1.02]	0.1886	0.981 [0.919–1.047]	0.5574
Osteopontin / +1000 pg/ml			0.994 [0.957–1.033]	0.7697	1.001 [0.962–1.04]	0.9794
Pentraxin-3 / +10 pg/ml			1.003 [0.972–1.035]	0.8548	0.981 [0.945–1.018]	0.3007
sTNFR1 / +1,000 pg/ml			1.784 [0.882–3.607]	0.1072	0.929 [0.617–1.4]	0.7255
sTNFR2 / +1,000 pg/ml			1.317 [0.969–1.79]	0.0785	0.79 [0.588–1.06]	0.1162
TWEAK/TNSF12 / +10 pg/ml			1.03 [0.955–1.112]	0.4429	0.983 [0.883–1.095]	0.7539
6Ckine/CCL21 / +1,000 pg/ml			1.104 [0.703–1.733]	0.6669	0.63 [0.366–1.086]	0.0964
BCA-1/CXCL13 / +10 pg/ml			0.984 [0.921–1.051]	0.6347	0.931 [0.802–1.081]	0.3501
CTACK/CCL27 / +100 pg/ml			1.001 [0.931–1.076]	0.9865	0.534 [0.287–0.991]	0.5596
ENA-78/CXCL5 / +100 pg/ml			1.052 [0.984–1.126]	0.1393	1.029 [0.977–1.083]	0.2799
Eotaxin/CCL11 / +10 pg/ml			0.964 [0.858–1.084]	0.5408	0.895 [0.767–1.043]	0.1556
Eotaxin-2/CCL24 / +100 pg/ml			1.036 [0.971–1.105]	0.2819	0.889 [0.811–0.974]	0.0117
RA: Fractalkine/CX3CL1 Tertile 1	SLE: Fractalkine/CX3CL1 / +100 pg/ml		0.467 [0.138–1.583]	0.2213		0.0466
RA: Fractalkine/CX3CL1 Tertile 2			0.52 [0.148–1.825]	0.3075
GCP-2/CXCL6 / +10 pg/ml			1.03 [0.881–1.204]	0.7122	0.971 [0.816–1.156]	0.743
GM-CSF / +10 pg/ml			1.065 [0.909–1.249]	0.4347	0.898 [0.753–1.071]	0.2329
RA: Gro-a/CXCL1 / +10 pg/ml	SLE: Gro-a/CXCL1 Tertile 1		1.018 [0.977–1.06]	0.3973	3.931 [0.901–17.155]	0.0686
	SLE: Gro-a/CXCL1 Tertile 2		1.663 [0.292–9.463]			0.5662
Gro-b/CXCL2 / +100 pg/ml			1.057 [0.938–1.192]	0.3636	0.965 [0.867–1.074]	0.5118
I-309/CCL1 / +10 pg/ml			1.025 [0.786–1.338]	0.8544	0.962 [0.728–1.272]	0.787
IL-4 / +10 pg/ml			0.623 [0.317–1.226]	0.1709	0.632 [0.293–1.363]	0.2422
IL-8 / +10 pg/ml			0.966 [0.883–1.056]	0.4444	0.904 [0.602–1.357]	0.6257
IL-16 / +100 pg/ml			1.027 [0.847–1.245]	0.7891	0.87 [0.645–1.175]	0.3641
IP-10/CXCL10 / +100 pg/ml			0.973 [0.781–1.213]	0.8105	0.808 [0.596–1.095]	0.1689
I-TAC/CXCL11 / +10 pg/ml			1.344 [1.027–1.76]	0.0314	0.932 [0.844–1.028]	0.1572
RA: MCP-1/CCL2 Tertile 1	SLE: MCP-1/CCL2 / +10 pg/ml		1.925 [0.568–6.519]	0.2927	0.839 [0.732–0.962]	0.0121
RA: MCP-1/CCL2 Tertile 2			2.2 [0.681–7.102]	0.1873		
MCP-2/CCL8 / +10 pg/ml			1.011 [0.883–1.157]	0.8734	0.92 [0.794–1.067]	0.2699
MCP-3/CCL7 / +10 pg/ml			0.935 [0.809–1.08]	0.3612	0.898 [0.738–1.094]	0.2856
MCP-4/CCL13 / +100 pg/ml			1.122 [0.579–2.175]	0.7337	0.712 [0.277–1.832]	0.4808
MDC/CCL22 / +100 pg/ml			1.013 [0.914–1.122]	0.8056	0.869 [0.749–1.008]	0.0633
MIF / +1000 pg/ml			0.942 [0.87–1.02]	0.141	1.329 [0.872–2.026]	0.1853
MIG/CXCL9 / +100 pg/ml			1.077 [0.962–1.206]	0.1965	0.956 [0.85–1.075]	0.4536
MIP-1a/CCL3 / +10 pg/ml			0.93 [0.778–1.111]	0.4215	0.601 [0.323–1.12]	0.1088
MIP-1d/CCL15 / +1,000 pg/ml			1.05 [0.985–1.119]	0.1313	0.963 [0.904–1.026]	0.24
MIP-3a/CCL20 / +10 pg/ml			0.977 [0.622–1.533]	0.9179	0.664 [0.355–1.244]	0.2013
RA: MIP-3b/CCL19 Classe 0	SLE: MIP-3b/CCL19 / +100 pg/ml		2.571 [0.361–18.325]	0.3459	0.928 [0.753–1.144]	0.4858
RA: MIP-3b/CCL19 Classes 1, 2, 3			0.429 [0.106–1.736]	0.2352		
RA: MIP-3b/CCL19 Classe 4			3.142 [0.45–21.952]	0.2483		
MPIF-1/CCL23 / +100 pg/ml			1.051 [0.82–1.348]	0.6944	0.887 [0.638–1.234]	0.4762
SCYB16/CXCL16 / +100 pg/ml			1.066 [0.848–1.341]	0.5843	0.934 [0.697–1.252]	0.6477
SDF-1a+b/CXCL12 / +1,000 pg/ml			0.954 [0.562–1.621]	0.8631	0.651 [0.345–1.23]	0.1864
RA: TARC/CCL17 Tertile 1	SLE: TARC/CCL17 / +100 pg/ml		0.706 [0.2–2.487]	0.5878	0.913 [0.678–1.23]	0.5507
RA: TARC/CCL17 Tertile 2			0.227 [0.065–0.793]	0.0202		
TECK/CCL25 / +100 pg/ml			1.002 [0.755–1.331]	0.9866	0.824 [0.566–1.2]	0.3125
TNFa / +10 pg/ml			0.798 [0.506–1.258]	0.3316	0.479 [0.247–0.928]	0.0292

Odds Ratios (OR), and their 95% confidence intervals (CI), for the likelihood of pSS diagnosis versus RA or SLE, in separate logistic regressions for each biomarker, are represented. ORs comparing pSS versus SLE were age-adjusted. For biomarkers specified as categorical variables in the models, the reference level was the highest class.

The serum concentration of four of the 63 tested biomarkers could statistically discriminate pSS patients from RA patients. A 10,000 pg/ml greater concentration of BDNF was significantly associated with a decreased likelihood of pSS diagnosis compared to RA (OR = 0.493 and 95% CI [0.273–0.891]; p = 0.0193). While greater concentrations of haptoglobin (10,000 ng/ml) and I-TAC/CXCL11 (10 pg/ml) were significantly associated with moderate increases in the likelihood of pSS diagnosis over RA (haptoglobin: OR = 1.015 and 95% CI [1.002–1,029]; p = 0.0262; I-TAC/CXCL11: OR = 1.344 and 95% CI [1.027–1.76]; p = 0.0314). TARC/CCL17 concentration in the second tertile, compared to the third tertile, was associated with a decreased likelihood of pSS diagnosis compared to RA (OR = 0.227 and 95% CI [0.065–0.793]; p = 0.0202). However, a lower concentration of this biomarker (first tertile) did not significantly alter the likelihood of pSS diagnosis, suggesting a potentially spurious association.

### Biomarkers Associated With pSS *Versus* SLE

Serum concentrations of eight biomarkers could statistically discriminate samples from pSS versus SLE patients (**Table 2**). A 100 ng/ml greater concentration of HDP was associated with a moderate decrease in the likelihood of pSS compared to SLE (OR = 0.9 and 95% CI [0.817–0.991]; p = 0.0318). CTSS concentration in classes 3 and 4, rather than in class 5, was strongly associated with an increased likelihood of pSS compared to SLE (OR = 14.064 and 95% CI [1.56–126.806]; p = 0.0185). However, lower concentrations of this marker (in classes 0, 1, or 2) could not significantly distinguish pSS from SLE. Similarly, SPARC concentration in class 4, rather than class 5, was associated with an increased likelihood of pSS compared to SLE (OR = 0.035 and 95% CI [0.002–0.66]; p = 0.0253), while at lower concentrations (in classes 0, 1, 2, or 3) there were no significant differences between the two AIDs. A 10,000 pg/ml greater concentration of sCD163 was significantly associated with a moderate decrease in the likelihood of pSS compared to SLE diagnosis (OR = 0.803 and 95% CI [0.649–0.994]; p = 0.0436). A 100 pg/ml greater concentration of Eotaxin-2/CCL24 was associated with a moderate decrease in the likelihood of pSS compared to SLE (OR = 0.889 and 95% CI [0.811–0.974]; p = 0.0117). A 100 pg/ml greater concentration of Fractalkine/CX3CL1 almost halved the likelihood of pSS compared to SLE (OR = 0.534 and 95% CI [0.287–0.991]; p = 0.0466). Lastly, a 10 pg/ml greater concentration of MCP-1/CCL2 was associated with a moderate decrease in the likelihood of pSS compared to SLE (OR = 0.839 and 95% CI [0.732–0.962]; p = 0.0121), while a 10 pg/ml greater concentration of TNFa was strongly associated with a decreased likelihood of pSS compared to SLE diagnosis (OR = 0.479 and 95% CI [0.247–0.928]; p = 0.0292).

### Specific Proteomic Signature in pSS

None of the studied biomarkers could simultaneously discriminate pSS from RA and SLE. We therefore determined the positive predictive value (PPV), sensitivity, and specificity of different combinations of BDNF, I-TAC/CXCL11, sCD163 and Fractalkine/CX3CL1 concentrations. These biomarkers were chosen because they were those most strongly associated with distinguishing pSS from the other AIDs (OR < 0.8 or OR > 1.2, and p<0.05), thus constituting a specific proteomic signature of pSS compared to RA and SLE (**Table 3**). Concentrations of these biomarkers were considered low or high based on their median concentration in the whole cohort. We observed that a combination of low BDNF and high Fractalkine concentrations was very specific for pSS (96.2%) with a PPV of 80% but weakly sensitive (19%). Conversely, a combination of high I-TAC concentration and low sCD163 concentration was strongly specific for pSS (98.1%), with a PPV of 75%, but with very low sensitivity (7.1%). Other biomarkers associations had significantly lower statistical performance (**Table 3**).

**Table 3:** Statistical performance of biomarker combinations to discriminate primary Sjögren’s syndrome (pSS) from rheumatoid arthritis (RA) and systemic lupus erythematosus (SLE).

	Low BDNF	HighI-TAC	Low sCD163		Low Fractalkine	pSS patients (n)	No-pSS patients (n)	PPV % (pSS)	Se % (pSS)	Spe % (pSS)
2 BMKs	X	X				2	3	40.0	4.8	94.3
	X			X		3	4	42.9	7.1	92.5
	X				X	8	2	80.0	19.0	96.2
		X		X		3	1	75.0	7.1	98.1
		X			X	1	3	25.0	2.4	94.3
				X	X	2	6	25.0	4.8	88.7
3 BMKs	X	X		X		4	2	66.7	9.5	96.2
	X	X			X	1	2	33.3	2.4	96.2
	X			X	X	4	8	33.3	9.5	84.9
		X		X	X	0	0	–	–	–
4 BMKs	X	X		X	X	0	1	0	0	98.1

Estimation of the positive predictive value (PPV), sensitivity (Se), and specificity (Spe) of different biomarker (BMKs) combinations to discriminate pSS (primary Sjogren Syndrome) from RA (Rheumatoid Arthritis) and SLE (Systemic Lupus Erythematosus). In contrast to other biomarkers, BDNF, I-TAC, sCD163 and Fractalkine serum concentrations were significantly associated with pSS diagnosis compared to RA or SLE (OR < 0.8 or > 1.2). Concentrations of these biomarkers were considered high or low relative to their median concentration in the whole cohort.

In addition, we investigated the association between expression of the four main biomarkers (BDNF, I-TAC/CXCL11, sCD163 and Fractalkine/CX3CL1) and disease activity (**Table S4, Supplemental Data**). A significant negative correlation was observed between pSS activity according to the ESSDAI score and serum sCD163 concentrations (r = −0.33859; p = 0.0283). In the SLE group, SLEDAI disease activity was significantly and positively correlated with serum BDNF concentrations (r = 0.49541; p = 0.0138). For the other biomarkers, there was no statistically significant correlation between their serum concentration and pSS, RA or SLE disease activity.

### Regulatory Roles of the Identified Biomarkers and Pathways Involved

**Figure 2:**
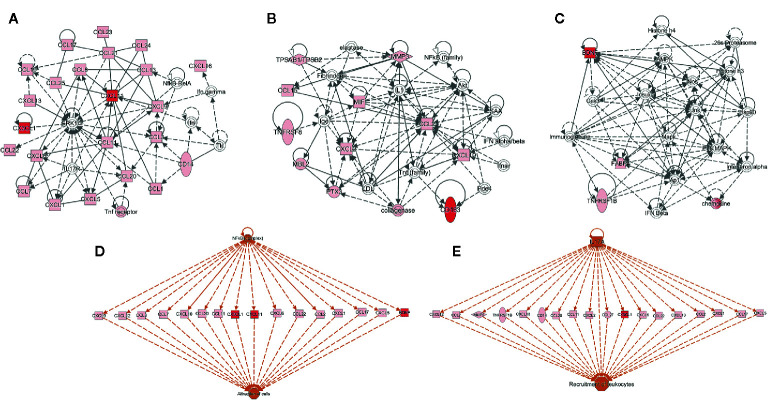
Functional and upstream regulatory networks. The most significant networks included proteins related to: **(A)** Cell-to-cell signaling and interaction, and cellular movement, **(B)** immune cell trafficking and inflammatory response, **(C)** organismal functions, hematological disease, and metabolic disease, **(D, E)** top-scoring network of regulator effects activated in the list of 63 biomarkers. Protein interaction networks were constructed using the ingenuity pathway analysis (IPA) software. Nodes shaded in pink represent proteins present in the list of 63 biomarkers; red nodes are proteins that are CXCL11 (I-TAC), CX3CL1 (Fractalkine), Brain Derived Neurotrophic Factor (BDNF), and sCD163; and proteins that are not found in the list of 63 biomarkers are in white. Dotted lines indicate indirect molecular interactions between proteins and continuous lines between nodes indicate direct functional interactions between connected proteins. Orange lines lead to activation.

Using IPA, we identified the top canonical pathways among the list of the 63 biomarkers that were enriched including BDNF, I-TAC/CXCL11, sCD163, and Fractalkine/CX3CL1. **Figure 2** displays the top networks found to be enriched in this list. Each network shows interactions *via* major signaling pathway proteins, including Extracellular signal-Regulated Kinases 1/2 (ERK1/2), Nuclear Factor-kappa B (NF-kB), IL-17, and interferon. Functions associated with the top networks include cellular movement, cell interaction, and inflammatory response. The I-TAC/CXCL11-centered network displayed a functional interaction with several cytokines and forming a tightly connected network with ERK1/2 (**Figure 2A**). Fractalkine/CX3CL1 was also related to ERK1/2 and displayed direct interaction with this pathway. Moreover, the network (**Figure 2B**) shows interactions between sCD163 and the TNF family, suggesting an operative role of TNF signaling pathways. In addition, BDNF displayed a tightly connected network with functional interaction with immunoglobulins, histones, and 26s proteasomes (**Figure 2C**). Finally, the upstream regulator analysis in IPA identified NF-kB and IL17A as key regulators of I-TAC/CXCL11, Fractalkine/CX3CL1, BDNF, and multiple cytokines that are implicated in the attraction of cells and recruitment of leukocytes (**Figures 2D, E**).

## Discussion

Our study compared the serum concentration of 63 biomarkers in patients with an established diagnosis of pSS to patients with established RA or SLE without secondary SS. We observed that differences in the concentration of BDNF, haptoglobin, and I-TAC/CXCL11 may allow discrimination of pSS from RA patients. Similarly, differences in the concentration of HDP, sCD163, Eotaxin-2/CCL24, Fractalkine/CX3CL1, MCP-1/CCL2, and TNFa may discriminate pSS from SLE. In addition, we observed that combinations of low BDNF and high Fractalkine/CX3CL1 concentrations, as well as high I-TAC and low sCD163 concentrations, were associated with high PPV and specificity for pSS. These four biomarkers may constitute a new and potentially useful specific signature for distinguishing pSS compared to RA and SLE. Indeed, our approach of screening a large number of biomarkers was also intended to highlight new lines of research, which could improve knowledge of pSS physiopathology, may differentiate pSS patients from patients with SLE or RA, and may provide future therapeutic targets.

Pathway analysis in which BDNF, Fractalkine/CX3CL1, and I-TAC/CXCL11 are linked strongly implies a potential functional role of NF-kB and IL-17 signaling pathways (**Figure 2**). Our results are consistent with previous reports of NF-kB pathway involvement in pSS pathophysiology. Indeed, its expression is increased in human salivary glands cells from pSS patients compared to controls (30). Polymorphisms of NF-kB target or regulatory genes are also associated with increased susceptibility to pSS (31) and pSS-related lymphoma (32–34). In addition, activation of the NF-kB pathway in Peripheral Mononuclear Blood Cells (PMBCs) from pSS patients may lead to IL-17 production through Toll-Like Receptor 2 (TLR2) activation (35), and NF-kB activation by TLR9 signaling appears to be facilitated in B lymphocytes of pSS patients (36). Therefore, the NF-kB pathway might be crucial in pSS pathophysiology through its activation in innate or acquired immunity cells, or even in epithelial cells, leading to an inflammatory response and activation of other inflammatory pathways such as IL-17, which is also involved in pSS development and progression (37).

To the best of our knowledge, our study is the first to compare serum BDNF levels in patients with pSS to those in patients with RA and SLE. Changes in BDNF concentration may depend on age or platelet levels (38). However, these two parameters were similar between the pSS and RA groups. Our study, therefore, suggests a different involvement of BDNF in pSS and RA physiopathology, either by its role in the nervous system (39), where BDNF is involved in proliferation and survival of nervous tissue (40) or by its action on the immune system (41) where BDNF affects the proliferation of T- and B-lymphocytes (42, 43).

I-TAC/CXCL11 is an IFNg-inducible chemokine, as are CXCL9 and CXCL10 (44), whose main function is to attract CXCR3-expressing cells (mainly activated T-lymphocytes, Natural Killer lymphocytes, and monocytes/macrophages) to the inflammatory site. Although CXCL11 seems to be involved in pSS salivary gland lesions (45–47), we did not find previous data regarding circulating CXCL11 in pSS patients. Furthermore, while serum concentrations of CXCL9 and CXCL10, two IFNg-induced chemokines able to bind CXCR3, were correlated with CXCL11 exclusively in the pSS group (p<0.0001), they could not discriminate pSS from RA or SLE (see **Table 2**). Therefore, it is possible that among the three ligands of CXCR3, only CXCL11 is differentially regulated in both RA and pSS, highlighting the role of IFNg in pSS.

sCD163 is derived from proteolytic cleavage of the extracellular portion of CD163 (48, 49), which is a membrane receptor found on anti-inflammatory M2 macrophages. Although sCD163 possesses anti-inflammatory activity (50), it is also considered a macrophage activation marker (51–54). Here, an increase in serum sCD163 concentration was significantly associated with a decreased likelihood of pSS compared to SLE. Corticosteroid could increase CD163^+^ cell counts and impact sCD163 circulating levels, although this has not yet been demonstrated (55). Here, the number of patients receiving corticosteroid was comparable between groups. However, the action of synthetic DMARDs or biologic drugs on sCD163 concentrations remains to be studied (55). Furthermore, sCD163 concentration may be associated with cardiovascular disease risk (56, 57). However, we did not find differences between SLE and pSS groups concerning the number of cardiovascular comorbidities. Thus, from a pathophysiological perspective, our results suggest either a different role of CD163+ macrophage lineage or a divergent regulation of sCD163 production in pSS and SLE.

Another molecule differentiating pSS from SLE is Fractalkine/CX3CL1, the latter being a membrane form chemokine released by proteolytic cleavage (58). In addition to Fractalkine’s chemotactic activity on monocytes and T-lymphocytes, it can facilitate the extravasation of leukocytes toward the inflammatory site (59). Fractalkine seems particularly involved in the pathophysiology of Sjogren sialadenitis (60–62) and lupus nephritis (63–65). Here, we observed an increase in serum Fractalkine concentration approximately halved the probability of pSS compared to SLE. We found only one study that analyzed serum Fractalkine concentration in SLE, RA, and pSS patients, with results consistent with ours (66). Fractalkine could therefore be involved in pSS and SLE pathophysiology, but with different regulation patterns and degrees of importance. As with I-TAC/CXCL11, Fractalkine is a chemokine induced by IFNg, which further emphasizes the primordial role of IFNg in pSS physiopathology. In addition, Lee et al. (61) found a significant correlation between serum concentrations of Fractalkine and TNFa in pSS patients. Here, serum concentrations of Fractalkine and TNFa were highly correlated in all groups (pSS: r = 0.689 and p<0.0001; RA: r = 0.608 and p = 0.0005; SLE: r = 0.563 and p = 0.003; data not shown), suggesting a strong pathophysiological relationship between these two biomarkers. TNFa is a pro-inflammatory cytokine involved in many autoimmune or auto-inflammatory diseases such as RA (67). While TNFa may be involved in the development of pSS in murine models (68), TNF inhibitors did not show efficacy in the treatment of pSS symptoms in humans (69–71). Thus, this cytokine may not be a key player in pSS pathophysiology. However, TNFa could be involved in SLE physiopathology, although its specific role remains to be specified (72, 73); our results are consistent with such a role of TNFa as we observed that greater serum concentrations of TNFa decreased the likelihood of pSS compared to SLE. Based on our data and those available in the literature, integration of serum TNFa concentration into a strategy to discriminate pSS from SLE appears premature.

Finally, we found that variation in serum concentrations of haptoglobin, HDP, MCP-1/CCL2, and Eotaxin-2/CCL24, were weakly associated with the likelihood of pSS compared to RA or SLE (0.8 < OR < 1.2). Despite statistically significant results, it is unlikely that these biomarkers could provide any clinical benefit. Similarly, we observed that at least one of the TARC/CCL17, CTSS, and SPARC concentration classes statistically discriminated pSS from one of the other AIDs. However, since pSS probability does not vary linearly across concentration classes, and given the small number of patients in SLE and RA groups, it is difficult to reach strong conclusions on these biomarkers’ discriminative abilities.

Our study has several limitations. First, heterogeneity in patient characteristics between each group could potentially influence the serum concentrations of the different biomarkers. For example, SLE patients were significantly younger than pSS and RA patients, disease duration was shorter in pSS group (and also in the chronic phase), and treatments differed among groups, particularly for biologic drugs. The potential confounding bias of age was corrected in logistic regression when necessary. Furthermore, the effect of immunomodulatory therapies on serum biomarker concentrations remains to be identified. We performed additional statistical analysis and the concomitant use of biologic drugs was not associated with significant changes in the expression of the four main biomarkers (BDNF, Fractalkine/CX3CL1, I-TAC/CXCL11 and sCD163). In addition, we did not find significant variations between patients with and without steroids except for the sCD163 biomarker where we observed lower levels in steroid-treated patients (**Table S5, Supplemental Data**). Another potential limitation is a lack of statistical power arising from the relatively small number of patients in RA and SLE groups, with sample sizes further reduced in some analyses by the exclusion of patients with no exploitable biological data (biomarker concentration outside the kit detection capabilities or variation of more than 20% between technical duplicates, **Table S2 Supplemental Data**). However, the number of patients does not seem so low when compared to previous studies recently published in the field (<100 patients) (21–23). Indeed, our results need to be reproduced and validated in a larger and independent cohort as suggested by Chau et al. (74), since only few of our candidate biomarkers may prove relevant, and we did not correct the alpha risk for multiple analyses. Finally, approximately 70% of our patients had a low disease activity, while a small minority of patients had a high disease activity (none in the SLE group). This restricted disease activity spectrum ensured a good comparison between groups. However, having a larger number of patients with a higher level of disease activity would probably improve biomarker identification and discriminatory ability.

In conclusion, serum concentrations of 63 immunological biomarkers in pSS, RA, and SLE patients showed that four biomarkers were relevant to discriminate pSS from RA (BDNF and I-TAC/CXCL11), and pSS from SLE (sCD163, Fractalkine/CX3CL1). The next steps are to validate the markers with a larger and more homogeneous population and then investigate how these four molecules contribute to the physiopathology of pSS. The NF-kB, IL-17, and interferon pathways seem to be implicated, consistent with the mechanisms already known in pSS.

## Data Availability Statement

The data sets presented in this study can be found in online repositories. The names of the repository/repositories and accession number(s) can be found in the article/**Supplementary Material**.

## Ethics Statement

The studies involving human participants were reviewed and approved by Comité de Protection des Personnes Sud Méditerranée IV: DC-2015-2584. The patients/participants provided their written informed consent to participate in this study.

## Author Contributions

CJ, PGa, and Y-MP designed the experiments. RF, PGu, AM, RG, CJ, and Y-MP collected clinical data. Experimental works were performed by PGa and SA, and data were analyzed by GP, CD, MJ, SA, and Y-MP. GP and Y-MP prepared the manuscript. All authors have contributed to revising the manuscript and final approval. All authors contributed to the article and approved the submitted version.

## Conflict of Interest

PGa was employed by company BioRad.

The remaining authors declare that the research was conducted in the absence of any commercial or financial relationships that could be construed as a potential conflict of interest.
