# Supplementary material for: Identification of a Novel Serum Proteomic Signature for Primary Sjögren’s Syndrome
Source: Front Immunol. 2021 Feb 23;12:631539. doi: 10.3389/fimmu.2021.631539 (PMC7942395; doi:10.3389/fimmu.2021.631539)
Supplement: Supplementary file 1 [file Table_1.docx]

Supplementary Material

**Table S1: Serum biomarker concentrations in the whole cohort and in pSS, RA and SLE groups.**

Table of mean, median and minimum/maximum concentrations of biomarkers in the total study population and in pSS, RA and SLE groups.

n corresponds to the number of patients included in the statistical analysis (after exclusion of samples with a CV > 20% between the two technical duplicates or with values outside the kit detection limits).

|  |  |  |  |  |  |
| --- | --- | --- | --- | --- | --- |
| **Biomarker** | **Parameters** | **Total Population** | **pSS** | **RA** | **SLE** |
| **SPP1 (ng/ml)** | Patients analyzed | n=95 | n=42 | n=28 | n=25 |
|  | Mean (±SD) | 33.14 (± 25.18) | 36.12 (± 34.11) | 31.64 (± 15.10) | 29.8 (± 14.47) |
|  | Median (Q1; Q3) | 28 (20; 37) | 28 (18; 37) | 25.5 (21; 42.5) | 28 (22; 33) |
|  | Median (min; max) | 28 (12; 200) | 28 (12; 200) | 25.5 (12; 80) | 28 (13; 80) |
| **HDP (ng/ml)** | Patients analyzed | n=94 | n=41 | n=28 | n=25 |
|  | Mean (±SD) | 1367.8 (± 757.09) | 1445.83 (± 655.05) | 1233.21 (± 620.46) | 1390.56 (± 1020.18) |
|  | Median (Q1; Q3) | 1145.5 (843; 1584) | 1387 (936; 1637) | 1101 (835.5; 1499.5) | 1048 (780; 1690) |
|  | Median (min; max) | 1145.5 (167; 4002) | 1387 (619; 3107) | 1101 (167; 3035) | 1048 (249; 4002) |
| **TPS2 (ng/ml)** | Patients analyzed | n=94 | n=42 | n=27 | n=25 |
|  | Mean (±SD) | 32.04 (± 12.44) | 31.67 (± 13.33) | 30.78 (± 11.62) | 34.04 (± 11.98) |
|  | Median (Q1; Q3) | 30 (25; 37) | 29 (25; 34) | 28 (24; 35) | 32 (30; 40) |
|  | Median (min; max) | 30 (9; 78) | 29 (9; 78) | 28 (10; 61) | 32 (15; 57) |
| **preHp2 (ng/ml)** | Patients analyzed | n=83 | n=40 | n=22 | n=21 |
|  | Mean (±SD) | 348.53 (± 342.48) | 357.45 (± 369.39) | 334.64 (± 331.72) | 346.1 (± 314.51) |
|  | Median (Q1; Q3) | 131 (68; 694) | 130 (74; 756.5) | 107.5 (67; 622) | 212 (80; 569) |
|  | Median (min; max) | 131 (34; 1072) | 130 (35; 1034) | 107.5 (38; 946) | 212 (34; 1072) |
| **LGalS3P (ng/ml)** | Patients analyzed | n=86 | n=38 | n=26 | n=22 |
|  | Mean (±SD) | 8214.63 (± 4481.9) | 8954.21 (± 4958.94) | 6936.15 (± 3751.44) | 8448.09 (± 4260.84) |
|  | Median (Q1; Q3) | 7269 (4917; 10177) | 7428.5 (5123; 10916) | 6309.5 (3910; 8676) | 8230 (5249; 10485) |
|  | Median (min; max) | 7269 (1517; 20001) | 7428.5 (3257; 20001) | 6309.5 (2173; 18843) | 8230 (1517; 17387) |

| **Adiponectin (ng/ml)** | Patients analyzed | n=95 | n=42 | n=28 | n=25 |
| --- | --- | --- | --- | --- | --- |
|  | Mean (±SD) | 11227.81 (± 5301.39) | 10812.52 (± 5081.4) | 12093.61 (± 5490.50) | 10955.8 (± 5549.73) |
|  | Median (Q1; Q3) | 10401 (7225; 14403) | 9587.5 (6348; 13744) | 11034 (9107; 14007) | 8988 (6034; 15629) |
|  | Median (min; max) | 10401 (3553; 31409) | 9587.5 (3774; 22167) | 11034.5 (5023; 31409) | 8988 (3553; 22090) |
| **oxLDL (mU/ml)** | Patients analyzed | n=95 | n=42 | n=28 | n=25 |
|  | Mean (±SD) | 68105.81 (± 22196.06) | 72162.45 (± 23056.02) | 70685.39 (± 18410.17) | 58401.52 (± 22483.98) |
|  | Median (Q1; Q3) | 63748 (52561; 82232) | 66658 (56661; 83601) | 67922 (59050; 85781.5) | 53997 (40273; 67987) |
|  | Median (min; max) | 63748 (24903; 137781) | 66658 (31247; 137781) | 67922 (41380; 119460) | 53997 (24903; 108950) |
| **HA (ng/ml)** | Patients analyzed | n=95 | n=42 | n=28 | n=25 |
|  | Mean (±SD) | 69.17 (± 58.12) | 65.33 (± 48.55) | 91.11 (± 79.12) | 51.04 (± 35.09) |
|  | Median (Q1; Q3) | 50 (36; 82) | 48 (39; 77) | 62 (48.5 ; 99.5) | 43 (24; 57) |
|  | Median (min; max) | 50 (13; 320) | 48 (13; 221) | 62 (14; 320) | 43 (19; 155) |
| **FABP4 (ng/ml)** | Patients analyzed | n=95 | n=42 | n=28 | n=25 |
|  | Mean (±SD) | 38.8 (± 34.65) | 43.81 (± 43.76) | 39.61 (± 23.82) | 29.48 (± 25.67) |
|  | Median (Q1; Q3) | 30 (20; 44) | 30 (24; 45) | 35.5 (23.5; 46.5) | 20 (14; 34) |
|  | Median (min; max) | 30 (8; 204) | 30 (11; 204) | 35.5 (13; 109) | 20 (8; 119) |
| **CTSS (pg/ml)** | Patients analyzed | n=95 | n=42 | n=28 | n=25 |
|  | Mean (±SD) | 11025.47 (± 2486.17) | 10980.6 (± 2227.94) | 11357.57 (± 2804.88) | 10728.92 (± 2579.69) |
|  | Median (Q1; Q3) | 10714 (9502; 12548) | 10741.5 (9841; 12506) | 11048 (10097; 13005) | 10229 (8906; 12548) |
|  | Median (min; max) | 10714 (4405; 18131) | 10741.5 (7093; 18023) | 11048 (4405; 18131) | 10229 (6805; 16222) |
| **BDNF (pg/ml)** | Patients analyzed | n=93 | n=41 | n=28 | n=24 |
|  | Mean (±SD) | 18342.02 (± 9461.33) | 16987.24 (± 8372.80) | 22554.39 (± 9871.52) | 15742 (± 9477.65) |
|  | Median (Q1; Q3) | 19679 (13139; 23825) | 16862 (13139; 23254) | 22109 (17513; 29755) | 18120.5 (6472; 21837) |
|  | Median (min; max) | 19679 (1206; 41394) | 16862 (1206; 30497) | 22109.5 (2157; 41394) | 18120.5 (2378; 40503) |
| **SPARC (ng/ml)** | Patients analyzed | n=91 | n=40 | n=28 | n=23 |
|  | Mean (±SD) | 613.84 (± 250.39) | 649.23 (± 286.26) | 609.79 (± 200.96) | 557.22 (± 236.63) |
|  | Median (Q1; Q3) | 631 (442; 768) | 650.5 (450; 817.5) | 649.5 (447; 762) | 532 (428; 758) |
|  | Median (min; max) | 631 (78; 1307) | 650.5 (78; 1307) | 649.5 (189; 945) | 532 (81; 1104) |

| **Haptoglobin (mg/ml)** | Patients analyzed | n=83 | n=40 | n=19 | n=24 |
| --- | --- | --- | --- | --- | --- |
|  | Mean (±SD) | 1177.7 (± 551.33) | 1333 (± 505.9) | 1005.7 (± 456.1) | 1055 (± 636.3) |
|  | Median (Q1; Q3) | 1101.8 (777; 1507) | 1205.8 (955.6; 1634.3) | 922.1 (640.8; 1459.4) | 932.2 (564.8; 1286.2) |
|  | Median (min; max) | 1101.9 (309; 3062) | 1205.8 (552; 2724) | 922.1 (309; 1794.8) | 932.2 (384.6; 3061.8) |
| **sCD14 (ng/ml)** | Patients analyzed | n=95 | n=42 | n=28 | n=25 |
|  | Mean (±SD) | 1583.3(± 481.327) | 1581.4 (± 401.3) | 1484 (± 322.8) | 1697.8 (± 699.9) |
|  | Median (Q1; Q3) | 1591.6 (1340.8; 1806.1) | 1597.1 (1400.8; 1821.8) | 1561.8 (1359.8; 1717.9) | 1619.6 (1300.3; 2067.2) |
|  | Median (min; max) | 1591.6 (242.1; 3781.2) | 1597.1 (352.7; 2849.1) | 1561.8 (544.7; 1836.7) | 1619.6 (242.1; 3781.2) |
| **MBL2 (ng/ml)** | Patients analyzed | n=88 | n=39 | n=27 | n=22 |
|  | Mean (±SD) | 1158.1 (± 889.4) | 1311.3 (± 979.7) | 1042 (± 938.7) | 1029.1 (± 609) |
|  | Median (Q1; Q3) | 905 (506; 1661.5) | 1095 (577; 1866) | 744 (358; 1369) | 1085.5 (467; 1395) |
|  | Median (min; max) | 905 (89; 4249) | 1095 (141 ; 4249) | 744 (149; 3783) | 1085.5 (89; 2268) |
| **APRIL/TNFSF13 (ng/ml)** | Patients analyzed | n=89 | n=39 | n=27 | n=23 |
|  | Mean (±SD) | 148.2 (± 101.3) | 148.1 (± 58.1) | 134.1 (± 43.3) | 165.1 (± 180.3) |
|  | Median (Q1; Q3) | 130.2 (103.2; 163.6) | 134.8 (106.4; 163.6) | 124.3 (100.7; 168.3) | 115.8 (91.8; 164.8) |
|  | Median (min; max) | 130.2 (71.9; 971.6) | 134.7 (89.7; 366.7) | 124.3 (71.9; 242.1) | 115.8 (75.8; 971.6) |
| **BAFF/TNFSF13B (pg/ml)** | Patients analyzed | n=95 | n=42 | n=28 | n=25 |
|  | Mean (±SD) | 17536.73 (± 20496.33) | 13241.55 (± 10199.59) | 20548.46 (± 22149.74) | 21379.48 (± 29282.8) |
|  | Median (Q1; Q3) | 10453 (8242; 14946) | 9425.5 (7887; 13389) | 10774 (8493; 20354) | 11789 (8385; 15150) |
|  | Median (min; max) | 10453 (4304; 141169) | 9425.5 (4304; 50031) | 10774 (6036; 94172) | 11789 (5998; 141169) |
| **sCD30/TNFRSF8 (pg/ml)** | Patients analyzed | n=94 | n=42 | n=27 | n=25 |
|  | Mean (±SD) | 529.67 (± 438.32) | 480.26 (± 262.63) | 457.3 (± 185.02) | 690.84 (± 743.21) |
|  | Median (Q1; Q3) | 429.5 (333; 585) | 421 (319; 569) | 392 (333; 585) | 462 (355; 756) |
|  | Median (min; max) | 429.5 (100; 3968) | 421 (100; 1778) | 392 (224; 827) | 462 (197; 3968) |
| **sCD163 (pg/ml)** | Patients analyzed | n=95 | n=42 | n=28 | n=25 |
|  | Mean (±SD) | 86383.61 (± 35192.43) | 89147.69 (± 29807.52) | 77408.96 (± 33775.14) | 91791.56 (± 43724.46) |
|  | Median (Q1; Q3) | 78569 (64491; 103024) | 87215 (72132; 99030) | 73788 (54231; 93107.5) | 74824 (63283; 107858) |
|  | Median (min; max) | 78569 (27626; 193069) | 87215 (29047; 193069) | 73788 (27626; 180408) | 74824 (39074; 189525) |

| **Chitinase 3-like 1 (pg/ml)** | Patients analyzed | n=95 | n=42 | n=28 | n=25 |
| --- | --- | --- | --- | --- | --- |
|  | Mean (±SD) | 19877.32 (± 9734) | 19968.88 (± 9388.14) | 22277.86 (± 8720.58) | 17034.88 (± 10946.75) |
|  | Median (Q1; Q3) | 18878 (11868; 24612) | 19326 (11868; 24612) | 21651 (16362.5; 28951) | 14825 (8505; 19194) |
|  | Median (min; max) | 18878 (5149; 53831) | 19326 (5149; 42742) | 21651 (8287; 42767) | 14825 (5535; 53831) |
| **gp130/sIL-6Rb (pg/ml)** | Patients analyzed | n=95 | n=42 | n=28 | n=25 |
|  | Mean (±SD) | 38341.74 (± 10601.44) | 40078.6 (± 10584.36) | 37124.46 (± 9546.69) | 36787.16 (± 11694.31) |
|  | Median (Q1; Q3) | 37807 (30671; 45115) | 39438 (33597; 46332) | 34847.5 (29785; 42236) | 34189 (28059; 44287) |
|  | Median (min; max) | 37807 (16693; 76991) | 39438 (19498; 76991) | 34847.5 (23949; 56300) | 34189 (16693; 65117) |
| **sIL-6Ra (pg/ml)** | Patients analyzed | n=95 | n=42 | n=28 | n=25 |
|  | Mean (±SD) | 13034.12 (± 5598.96) | 11914.86 (± 3131.20) | 14948.71 (± 8730.22) | 12770.12 (± 3707.65) |
|  | Median (Q1; Q3) | 12523 (9832; 14301) | 12365.5 (9832; 13628) | 12557.5 (10567.5; 15558.5) | 13548 (9618; 14705) |
|  | Median (min; max) | 12523 (4065; 49713) | 12365.5 (4065; 21041) | 12557.5 (7689; 49713) | 13548 (5916; 21392) |
| **MMP-2 (pg/ml)** | Patients analyzed | n=95 | n=42 | n=28 | n=25 |
|  | Mean (±SD) | 16602.82 (± 9977.5) | 18243.02 (± 12341.93) | 15692.61 (± 7464.99) | 14866.72 (± 7627.67) |
|  | Median (Q1; Q3) | 13541 (9754; 21515) | 14500.5 (10156; 21515) | 13977.5 (10580; 19310) | 12868 (8341; 21842) |
|  | Median (min; max) | 13541 (4208; 58423) | 14500.5 (5401; 58423) | 13977.5 (6010; 38454) | 12868 (4208; 29897) |
| **MMP-3 (pg/ml)** | Patients analyzed | n=82 | n=37 | n=25 | n=20 |
|  | Mean (±SD) | 5610.12 (± 4714.80) | 5617.51 (± 4566.67) | 5476.08 (± 4472.11) | 5764 (± 5471.93) |
|  | Median (Q1; Q3) | 3985.5 (2966; 5836) | 4152 (3203; 6139) | 3941 (2932; 5836) | 3722.5 (2921; 5113.50) |
|  | Median (min; max) | 3985.5 (1976; 24537) | 4152 (1976; 24537) | 3941 (2162; 23106) | 3722.5 (2090; 21971) |
| **Osteocalcin (pg/ml)** | Patients analyzed | n=82 | n=39 | n=20 | n=23 |
|  | Mean (±SD) | 2920.01 (± 4499.18) | 2324.33 (± 810.26) | 2657.5 (± 1081.09) | 4158.35 (± 8372.07) |
|  | Median (Q1; Q3) | 2346.5 (1770; 3009) | 2334 (1793; 2928) | 2628 (1707; 3261.50) | 2268 (1754; 3325) |
|  | Median (min; max) | 2346.5 (849; 42197) | 2334 (849; 4370) | 2628 (1085; 4613) | 2268 (1112; 42197) |
| **Osteopontin (pg/ml)** | Patients analyzed | n=95 | n=42 | n=28 | n=25 |
|  | Mean (±SD) | 23257.54 (± 14876) | 22323.67 (± 13806.20) | 23210.18 (± 10437.61) | 24879.48 (± 20353.91) |
|  | Median (Q1; Q3) | 19985 (12532; 29276) | 18264.5 (10949; 30540) | 22073(16280; 30531) | 20313 (16930; 25969) |
|  | Median (min; max) | 19985 (7060; 111453) | 18264.5 (7515; 66040) | 22073.5 (7060; 49422) | 20313 (7339; 111453) |

| **Pentraxin-3 (pg/ml)** | Patients analyzed | n=91 | n=40 | n=27 | n=24 |
| --- | --- | --- | --- | --- | --- |
|  | Mean (±SD) | 295.57 (± 163.43) | 299.03 (± 177.91) | 291.81 (± 129.81) | 294.04 (± 178.19) |
|  | Median (Q1; Q3) | 242 (177; 370) | 237.5 (192; 352) | 262 (186; 386) | 227.5 (156; 368) |
|  | Median (min; max) | 242 (101; 934) | 237.5 (103; 934) | 262 (111; 612) | 227.5 (101; 681) |
| **sTNF-R1 (pg/ml)** | Patients analyzed | n=94 | n=41 | n=28 | n=25 |
|  | Mean (±SD) | 2242.37 (± 1927.44) | 2229.07 (± 1042.42) | 1851.96 (± 644.96) | 2701.44 (± 3423.30) |
|  | Median (Q1; Q3) | 1824 (1500; 2455) | 1955 (1650; 2614) | 1725.5 (1493; 2216.50) | 1948 (1362; 2630) |
|  | Median (min; max) | 1824 (637; 18559) | 1955 (1106; 6289) | 1725.5 (781; 3878) | 1948 (637; 18559) |
| **sTNF-R2 (pg/ml)** | Patients analyzed | n=95 | n=42 | n=28 | n=25 |
|  | Mean (±SD) | 4556.79 (± 3652.75) | 4432.67 (± 2183.13) | 3550 (± 1522.85) | 5892.92 (± 6199.85) |
|  | Median (Q1; Q3) | 3626 (2827; 5378) | 3872.5 (3062; 5047) | 3298.5 (2246; 4785.50) | 4205 (2885; 6850) |
|  | Median (min; max) | 3626 (1169; 33317) | 3872.5 (1857; 11537) | 3298.5 (1169; 6797) | 4205 (1610; 33317) |
| **TWEAK/TNSF12 (pg/ml)** | Patients analyzed | n=95 | n=42 | n=28 | n=25 |
|  | Mean (±SD) | 170.46 (± 63.10) | 178.95 (± 70.69) | 166.75 (± 56.67) | 160.36 (± 56.52) |
|  | Median (Q1; Q3) | 159 (123; 211) | 160 (123; 227) | 155.5 (135; 190) | 146 (119; 230) |
|  | Median (min; max) | 159 (80; 402) | 160 (80; 402) | 155.5 (84; 321) | 146 (80; 246) |
| **6Ckine/CCL21 (pg/ml)** | Patients analyzed | n=95 | n=42 | n=28 | n=25 |
|  | Mean (±SD) | 4489.65 (± 2093.40) | 4203.17 (± 953.74) | 4090.32 (± 1262.08) | 5418.2 (± 3548.30) |
|  | Median (Q1; Q3) | 4144 (3554; 4783) | 3961 (3492; 4722) | 4130 (3419; 4515.50) | 4253 (3811; 5540) |
|  | Median (min; max) | 4144 (973; 20595) | 3961 (2103; 6461) | 4130 (973; 7485) | 4253 (2576; 20595) |
| **BCA-1/CXCL13 (pg/ml)** | Patients analyzed | n=95 | n=42 | n=28 | n=25 |
|  | Mean (±SD) | 53.48 (± 91.43) | 40.71 (± 38.53) | 49.29 (± 105.99) | 79.64 (± 128.63) |
|  | Median (Q1; Q3) | 26 (21; 42) | 26 (21; 39) | 26 (16.5; 33) | 32 (23; 52) |
|  | Median (min; max) | 26 (8 ; 573) | 26 (9; 196) | 26 (8; 573) | 32 (17; 528) |
| **CTACK/CCL27 (pg/ml)** | Patients analyzed | n=95 | n=42 | n=28 | n=25 |
|  | Mean (±SD) | 1725.29 (± 674.28) | 1732.31 (± 624.96) | 1729.57 (± 737.31) | 1708.72 (± 708.17) |
|  | Median (Q1; Q3) | 1689 (1284; 2119) | 1669.5 (1316; 2235) | 1750 (1237; 2040.5) | 1487 (1276; 2001) |
|  | Median (min; max) | 1689 (343; 3731) | 1669.5 (459; 2947) | 1750 (343; 3273) | 1487 (788; 3731) |

| **ENA-78/CXCL5 (pg/ml)** | Patients analyzed | n=73 | n=31 | n=23 | n=19 |
| --- | --- | --- | --- | --- | --- |
|  | Mean (±SD) | 1406.77 (± 1577.22) | 1624.74 (± 1822.58) | 968.30 (± 579.64) | 1581.89 (± 1908.85) |
|  | Median (Q1; Q3) | 906 (587; 1499) | 956 (618; 1710) | 794 (531; 1487) | 906 (537; 1725) |
|  | Median (min; max) | 906 (242; 8652) | 956 (430; 8652) | 794 (242; 2355) | 906 (268; 7445) |
| **Eotaxin/CCL11 (pg/ml)** | Patients analyzed | n=95 | n=42 | n=28 | n=25 |
|  | Mean (±SD) | 111.15 (± 43.66) | 109.12 (± 44.29) | 115.25 (± 36.80) | 109.96 (± 50.62) |
|  | Median (Q1; Q3) | 109 (81; 140) | 107.5 (74; 139) | 109 (89; 150) | 109 (78; 129) |
|  | Median (min; max) | 109 (32; 230) | 107.5 (32; 207) | 109 (48; 193) | 109 (35; 230) |
| **Eotaxin-2/CCL24 (pg/ml)** | Patients analyzed | n=94 | n=42 | n=27 | n=25 |
|  | Mean (±SD) | 1219.24 (± 890.18) | 1299.62 (± 884.26) | 1080.44 (± 705.03) | 1234.12 (± 1078.5) |
|  | Median (Q1; Q3) | 931 (616; 1507) | 1136 (652; 1548) | 825 (660; 1114) | 847 (595; 1810) |
|  | Median (min; max) | 931 (171; 4236) | 1136 (203; 4146) | 825 (171; 2852) | 847 (224; 4236) |
| **Fractalkine/CX3CL1 (pg/ml)** | Patients analyzed | n=95 | n=42 | n=28 | n=25 |
|  | Mean (±SD) | 335.77 (± 175.03) | 310.95 (± 130.89) | 307.79 (± 123.53) | 408.8 (± 256.91) |
|  | Median (Q1; Q3) | 292 (239; 392) | 296 (209; 381) | 255.5 (238.5; 334.5) | 334 (267; 409) |
|  | Median (min; max) | 292 (115; 1233) | 296 (115; 656) | 255.5 (173; 672) | 334 (168; 1233) |
| **GCP-2/CXCL6 (pg/ml)** | Patients analyzed | n=93 | n=42 | n=28 | n=23 |
|  | Mean (±SD) | 57.73 (± 31.77) | 57.19 (± 37.31) | 54.36 (± 20.95) | 62.83 (± 32.34) |
|  | Median (Q1; Q3) | 48 (37; 66) | 44.5 (33; 66) | 49.5 (39.5 ; 65.50) | 50 (40; 71) |
|  | Median (min; max) | 48 (18; 178) | 44.5 (18; 178) | 49.50 (22; 98) | 50 (27; 149) |
| **GM-CSF (pg/ml)** | Patients analyzed | n=75 | n=30 | n=25 | n=20 |
|  | Mean (±SD) | 93.52 (± 44.47) | 90.20 (± 39.24) | 82.96 (± 27.73) | 111.7 (± 62.21) |
|  | Median (Q1; Q3) | 87 (63; 112) | 94.50 (51; 112) | 79 (64; 104) | 95 (75.5; 131) |
|  | Median (min; max) | 87 (35; 284) | 94.50 (37; 170) | 79 (36; 138) | 95 (35; 284) |
| **Gro-a/CXCL1 (pg/ml)** | Patients analyzed | n=95 | n=42 | n=28 | n=25 |
|  | Mean (±SD) | 279.99 (± 164.13) | 291.19 (± 222.27) | 252.21 (± 57.87) | 292.28 (± 126.75) |
|  | Median (Q1; Q3) | 260 (203; 305) | 253.50 (197; 294) | 243 (206.5; 283) | 298 (229; 338) |
|  | Median (min; max) | 260 (96; 1514) | 253.50 (104; 1514) | 243 (163; 401) | 298 (96; 736) |

| **Gro-b/CXCL2 (pg/ml)** | Patients analyzed | n=95 | n=42 | n=28 | n=25 |
| --- | --- | --- | --- | --- | --- |
|  | Mean (±SD) | 621.53 (± 477.86) | 634.69 (± 582.32) | 522.54 (± 309.76) | 710.28 (± 432.63) |
|  | Median (Q1; Q3) | 516 (326; 742) | 504 (311; 727) | 435 (310; 657) | 543 (435; 1063) |
|  | Median (min; max) | 516 (98; 3455) | 504 (98; 3455) | 435 (142; 1395) | 543 (128; 1748) |
| **I-309/CCL1 (pg/ml)** | Patients analyzed | n=91 | n=39 | n=28 | n=24 |
|  | Mean (±SD) | 57.49 (± 22.38) | 56.49 (± 21.70) | 55.64 (± 13.80) | 61.29 (± 30.62) |
|  | Median (Q1; Q3) | 53 (46; 61) | 53 (46; 58) | 53 (45.5; 62) | 53 (49; 65) |
|  | Median (min; max) | 53 (35; 193) | 53 (37; 154) | 53 (35; 101) | 53 (36; 193) |
| **IL-4 (pg/ml)** | Patients analyzed | n=84 | n=36 | n=26 | n=22 |
|  | Mean (±SD) | 25.2 (± 10.40) | 22.61 (± 7.19) | 25.92 (± 10.40) | 28.59 (± 13.72) |
|  | Median (Q1; Q3) | 23 (19; 27) | 21 (18.5; 24.5) | 24 (19; 28) | 25.5 (21; 30) |
|  | Median min; max) | 23 (15; 72) | 21 (15; 58) | 24 (15; 66) | 25.5 (17; 72) |
| **IL-8/CXCL8 (pg/ml)** | Patients analyzed | n=89 | n=38 | n=28 | n=23 |
|  | Mean (±SD) | 31.12 (± 69.61) | 24.92 (± 13.73) | 43.07 (± 121.83) | 26.83 (± 24.06) |
|  | Median (Q1; Q3) | 20 (15; 26) | 20.50 (16; 28) | 20 (15; 24.50) | 18 (15; 30) |
|  | Median (min; max) | 20 (7; 664) | 20.50 (7; 68) | 20 (11; 664) | 18 (8; 122) |
| **IL-16 (pg/ml)** | Patients analyzed | n=95 | n=42 | n=28 | n=25 |
|  | Mean (±SD) | 729.09 (± 244.18) | 745.07 (± 251.03) | 728.79 (± 255.97) | 702.6 (± 225.82) |
|  | Median (Q1; Q3) | 688 (550; 853) | 681.5 (576; 850) | 724 (545; 895.50) | 663 (555; 782) |
|  | Median (min ; max) | 688 (225; 1466) | 681.5 (334; 1292) | 724 (225; 1256) | 663 (338; 1466) |
| **IP-10/CXCL10 (pg/ml)** | Patients analyzed | n=95 | n=42 | n=28 | n=25 |
|  | Mean (±SD) | 371.65 (± 246.54) | 356.12 (± 222.36) | 368.79 (± 215.02) | 400.96 (± 316.62) |
|  | Median (Q1; Q3) | 294 (191; 514) | 279.5 (181; 521) | 329 (189.5; 492) | 304 (192; 481) |
|  | Median (min; max) | 294 (44; 1378) | 279.5 (44; 898) | 329 (148; 1123) | 304 (68; 1378) |
| **I-TAC/CXCL11 (pg/ml)** | Patients analyzed | n=95 | n=42 | n=28 | n=25 |
|  | Mean (±SD) | 54.28 (± 135.54) | 45.38 (± 42.92) | 25.36 (± 14.31) | 101.64 (± 255.21) |
|  | Median (Q1; Q3) | 28 (17; 46) | 31 (19; 53) | 20.5 (14.5; 35.5) | 31 (21; 47) |
|  | Median (min; max) | 28 (5; 1275) | 31 (6; 224) | 20.5 (8; 55) | 31 (5; 1275) |

| **MCP-1/CCL2 (pg/ml)** | Patients analyzed | n=95 | n=42 | n=28 | n=25 |
| --- | --- | --- | --- | --- | --- |
|  | Mean (±SD) | 78.09 (± 47.35) | 73.62 (± 50.33) | 81.36 (± 41.93) | 81.96 (± 49.09) |
|  | Median (Q1; Q3) | 66 (44; 98) | 61 (41; 87) | 70 (48; 104) | 80 (40; 116) |
|  | Median (min; max) | 66 (18; 245) | 61 (18; 245) | 70 (38; 195) | 80 (18; 199) |
| **MCP-2/CCL8 (pg/ml)** | Patients analyzed | n=95 | n=42 | n=28 | n=25 |
|  | Mean (±SD) | 85.78 (± 42.47) | 81.95 (± 40.9) | 80.57 (± 27.04) | 98.04 (± 56.31) |
|  | Median (Q1; Q3) | 84 (56; 108) | 80.5 (48; 108) | 84.50 (60; 95) | 89 (61; 122) |
|  | Median (min; max) | 84 (6; 315) | 80.5 (6; 186) | 84.5 (34; 160) | 89 (36; 315) |
| **MCP-3/CCL7 (pg/ml)** | Patients analyzed | n=95 | n=42 | n=28 | n=25 |
|  | Mean (±SD) | 88.51 (± 34.2) | 85.45 (± 32.61) | 92.93 (± 35.11) | 88.68 (± 36.58) |
|  | Median (Q1; Q3) | 87 (61; 111) | 86 (53; 111) | 88.5 (66; 112) | 90 (63; 108) |
|  | Median (min; max) | 87 (27; 194) | 86 (29; 148) | 88.5 (43; 194) | 90 (27; 170) |
| **MCP-4/CCL13 (pg/ml)** | Patients analyzed | n=95 | n=42 | n=28 | n=25 |
|  | Mean (±SD) | 123.11 (± 70.2) | 129.52 (± 72.58) | 123.43 (± 76.91) | 111.96 (± 58.69) |
|  | Median (Q1; Q3) | 122 (77; 155) | 133 (77; 161) | 118.5 (72.5; 137) | 114 (77; 155) |
|  | Median (min; max) | 122 (16; 415) | 133 (19; 316) | 118.5 (18; 415) | 114 (16; 239) |
| **MDC/CCL22 (pg/ml)** | Patients analyzed | n=95 | n=42 | n=28 | n=25 |
|  | Mean (±SD) | 1108.82 (± 456.26) | 1077.9 (± 475.71) | 1049.68 (± 477.61) | 1227 (± 389.64) |
|  | Median (Q1; Q3) | 1081 (849; 1374) | 1044.5 (873; 1329) | 991.5 (769; 1275.5) | 1271 (1024; 1498) |
|  | Median (min; max) | 1081 (186; 2457) | 1044.5 (187; 2457) | 991.5 (186; 2126) | 1271 (523; 1939) |
| **MIF (pg/ml)** | Patients analyzed | n=86 | n=37 | n=26 | n=23 |
|  | Mean (±SD) | 4500.92 (± 8631.42) | 3709.11 (± 4396) | 8043.85 (± 14226.32) | 1769.65 (± 1596.73) |
|  | Median (Q1; Q3) | 1466.5 (843; 4481) | 1847 (829; 4963) | 1739.5 (1104; 9069) | 1185 (752; 2043) |
|  | Median (min; max) | 1466.5 (357; 66459) | 1847 (357; 20568) | 1739.5 (437; 66459) | 1185 (378; 6633) |
| **MIG/CXCL9 (pg/ml)** | Patients analyzed | n=95 | n=42 | n=28 | n=25 |
|  | Mean (±SD) | 708.04 (± 536.63) | 779.29 (± 626.49) | 609.89 (± 288.55) | 698.28 (± 586.08) |
|  | Median (Q1; Q3) | 561 (368; 821) | 607.5 (365; 833) | 546.5 (364; 707) | 481 (384; 798) |
|  | Median (min; max) | 561 (113; 3092) | 607.5 (128; 3092) | 546.5 (222; 1232) | 481 (113; 3063) |

| **MIP-1a/CCL3 (pg/ml)** | Patients analyzed | n=94 | n=42 | n=28 | n=24 |
| --- | --- | --- | --- | --- | --- |
|  | Mean (±SD) | 15.01 (± 43.44) | 10.19 (± 9.60) | 23.68 (± 78.50) | 13.33 (± 9.69) |
|  | Median (Q1; Q3) | 8 (7; 11) | 8 (7; 10) | 8 (7; 10.50) | 9 (7; 16) |
|  | Median (min; max) | 8 (5; 424) | 8 (5; 66) | 8 (6; 424) | 9 (5; 37) |
| **MIP-1d/CCL15 (pg/ml)** | Patients analyzed | n=91 | n=40 | n=27 | n=24 |
|  | Mean (±SD) | 11263.27 (± 9634.74) | 12473.13 (± 11974.32) | 8538.56 (± 5704.12) | 12312.17 (± 8421.49) |
|  | Median (Q1; Q3) | 8605 (4870; 14348) | 8853 (4499; 15244) | 7815 (3434; 11915) | 9076 (6157; 17707.50) |
|  | Median (min; max) | 8605 (408; 56706) | 8853 (417; 56706) | 7815 (408; 22646) | 9076 (3291; 34757) |
| **MIP-3a/CCL20 (pg/ml)** | Patients analyzed | n=67 | n=25 | n=22 | n=20 |
|  | Mean (±SD) | 23.28 (± 34.11) | 17.8 (± 10.76) | 18.18 (± 15.09) | 35.75 (± 58.40) |
|  | Median (Q1; Q3) | 13 (11; 21) | 13 (12; 20) | 13 (11; 19) | 20 (12; 26) |
|  | Median (min; max) | 13 (9; 271) | 13 (9; 49) | 13 (9; 81) | 20 (9; 271) |
| **MIP-3b/CCL19 (pg/ml)** | Patients analyzed | n=95 | n=42 | n=28 | n=25 |
|  | Mean (±SD) | 486.73 (± 374.67) | 487.67 (± 358.13) | 480.79 (± 414.71) | 491.8 (± 370.34) |
|  | Median (Q1; Q3) | 419 (234; 596) | 457 (201; 656) | 342.5 (242; 489.50) | 423 (240; 517) |
|  | Median (min; max) | 419 (98; 2000) | 457 (108; 1612) | 342.5 (139; 2000) | 423 (98; 1640) |
| **MPIF-1/CCL23 (pg/ml)** | Patients analyzed | n=94 | n=41 | n=28 | n=25 |
|  | Mean (±SD) | 363.41 (± 202.63) | 378.41 (± 195.18) | 359.68 (± 199.64) | 343 (± 223.45) |
|  | Median (Q1; Q3) | 354.5 (212; 482) | 375 (263; 504) | 343.5 (196; 493) | 346 (205; 454) |
|  | Median (min; max) | 354.5 (17; 898) | 375 (17; 898) | 343.5 (53; 806) | 346 (34; 867) |
| **SCYB16/CXCL16 (pg/ml)** | Patients analyzed | n=95 | n=42 | n=28 | n=25 |
|  | Mean (±SD) | 641.56 (± 210.05) | 643.88 (± 225.64) | 616.04 (± 185.64) | 666.24 (± 213.66) |
|  | Median (Q1; Q3) | 661 (523; 764) | 677 (526; 781) | 630.5 (518.5; 747) | 620 (549; 721) |
|  | Median (min; max) | 661 (69; 1434) | 677 (69; 1088) | 630.5 (113; 948) | 620 (409; 1434) |
| **SDF-1a+b/CXCL12 (pg/ml)** | Patients analyzed | n=95 | n=42 | n=28 | n=25 |
|  | Mean (±SD) | 3217.63 (± 978.58) | 3093.02 (± 908.40) | 3131 (± 925.92) | 3524 (± 1114.78) |
|  | Median (Q1; Q3) | 3196 (2609; 3812) | 3068.5 (2471; 3513) | 3236 (2497.5; 3776) | 3449 (2868; 3854) |
|  | Median (min; max) | 3196 (1123; 5773) | 3068.5 (1123; 5170) | 3236 (1356; 5041) | 3449 (1487; 5773) |

| **TARC/CCL17 (pg/ml)** | Patients analyzed | n=95 | n=42 | n=28 | n=25 |
| --- | --- | --- | --- | --- | --- |
|  | Mean (±SD) | 247.52 (± 244.97) | 265.98 (± 275.68) | 228.64 (± 225.02) | 237.64 (± 217.12) |
|  | Median (Q1; Q3) | 178 (99; 305) | 201.5 (85; 346) | 153.5 (112; 209.50) | 176 (99; 237) |
|  | Median (min; max) | 178 (31; 1522) | 201.5 (31; 1522) | 153.5 (52; 1052) | 176 (31; 879) |
| **TECK/CCL25 (pg/ml)** | Patients analyzed | n=95 | n=42 | n=28 | n=25 |
|  | Mean (±SD) | 511.67 (± 196.20) | 504.05 (± 186.41) | 503.36 (± 145.01) | 533.8 (± 258.97) |
|  | Median (Q1; Q3) | 509 (374; 594) | 523 (348; 639) | 491.5 (372.5; 588.50) | 538 (388; 575) |
|  | Median (min; max) | 509 (220; 1538) | 523 (220; 854) | 491.5 (232; 801) | 538 (237; 1538) |
| **TNF-a (pg/ml)** | Patients analyzed | n=94 | n=41 | n=28 | n=25 |
|  | Mean (±SD) | 32.27 (± 11.13) | 30.98 (± 10.22) | 33.54 (± 11.49) | 32.96 (± 12.33) |
|  | Median (Q1; Q3) | 31 (26; 39) | 31 (26; 37) | 32 (25.5; 38.50) | 33 (27; 40) |
|  | Median (min; max) | 31 (1; 63) | 31 (14; 59) | 32 (16; 63) | 33 (1; 54) |

**Table S2: Number of samples excluded from multivariate statistical analysis.**

Distribution of samples excluded from statistical analysis for each biomarker, according to the pattern: result outside the kit detection limits (upper or lower), or coefficient of variation > 20% between technical duplicates.

| **Biomarker** | **Below lower limit (n)** | **Above upper limit (n)** | **CV > 20% (n)** | **Total (n (%))** |
| --- | --- | --- | --- | --- |
| **TPS2** | 1 |  |  | 1 (1,1%) |
| **pre-Hp2** | 9 |  | 3 | 12 (12,6%) |
| **LGalS3P** |  | 1 | 8 | 9 (9,5%) |
| **BDNF** | 2 |  |  | 2 (2,1%) |
| **SPARC** | 4 |  |  | 4 (4,2%) |
| **Haptoglobin** | 6 |  | 6 | 12 (12,6%) |
| **MBL2** | 2 |  | 5 | 7 (7,4%) |
| **APRIL/TNFSF 13** | 1 |  | 5 | 6 (6,3%) |
| **sCD30** |  |  | 1 | 1 (1,1%) |
| **MMP-3** |  |  | 13 | 13 (13,7%) |
| **Osteocalcin** | 2 |  | 11 | 13 (13,7%) |
| **Pentraxin** |  |  | 4 | 4 (4,2%) |
| **sTNF-R1** |  |  | 1 | 1 (1,1%) |
| **ENA-78/CXCL5** | 18 |  | 4 | 22 (23,2%) |
| **Eotaxin-2/CCL24** | 1 |  |  | 1 (1,1%) |
| **GCP-2/CXCL6** | 1 |  | 2 | 3 (3,2%) |
| **GM-CSF** | 12 |  | 8 | 20 (21,1%) |
| **I-309/CCL1** |  |  | 4 | 4 (4,2%) |
| **IL-4** | 2 |  | 9 | 11 (11,6%) |
| **IL-8/CXCL8** | 1 |  | 5 | 6 (6,3%) |
| **MIF** | 4 |  | 5 | 9 (9,5%) |
| **MIP-1a/CCL3** | 1 |  |  | 1 (1,1%) |
| **MIP-1d/CCL15** |  | 4 |  | 4 (4,2%) |
| **MIP-3a/CCL20** | 3 |  | 25 | 28 (29,5%) |
| **MPIF-1/CCL23** |  |  | 1 | 1 (1,1%) |
| **TNF-a** |  |  | 1 | 1 (1,1%) |

**Table S3: Distribution of biological diagnostic criteria within different groups.**

Abbreviations: pSS: primary Sjogren Syndrome; ASGB: accessory salivary gland biopsy; RA: Rheumatoid Arthritis; RF: Rheumatoid Factor; ACPA: anti-citrullinated protein antibodies; SLE: Systemic Lupus Erythematosus; APS: anti-phospholipid syndrome.

| **A. pSS group** | **Patients (n=42)** | **%** |
| --- | --- | --- |
| Positive ASGB (focus > or = 1) | 36 | 85.7 |
| Positive Anti-SSa/b | 16 | 38.1 |
| Positive ASGB only | 24 | 57.1 |
| Positive Anti-SSa/b only | 4 | 9.5 |
| Positive ASGB and anti-SSa/b | 12 | 28.6 |

| **B. RA group** | **Patients (n=28)** | **%** |
| --- | --- | --- |
| Positive RF or ACPA | 20 | 71.4 |
| Erosion status | 14 | 50.0 |
| CRP > 5mg/l | 9 | 32.1 |

| **C. SLE group** | **Patients (n=25)** | **%** |
| --- | --- | --- |
| No auto-antibody | 1 | 4 |
| Anti-nuclear antibodies (isolated) | 4 | 16 |
| Anti-DNA | 15 | 60 |
| Anti-Sm | 3 | 12 |
| Anti-RNP | 4 | 16 |
| Anti-SSa (anti-SSa alone) | 8 (2) | 32 (8) |
| Associated biological APS | 6 | 24 |

**Table S4: Correlation between concentrations of significant biomarkers and disease activity of pSS, RA, or SLE**

Results of Spearman correlation test between the significant biomarkers concentrations and disease activity of pSS (according to ESSDAI score), RA (according to DAS28-CRP score) or SLE (according to SLEDAI score).

Abbreviations: r: Spearman's correlation coefficient; p: p-value; n: number of patients included in the biomarker analysis.

| **Biomarker** | **Parameter** | **pSS (ESSDAI)** | | **PR (DAS28-CRP)** | **LES (SLEDAI)** |
| --- | --- | --- | --- | --- | --- |
| **BDNF** | r | | 0.05762 | -0,06522 | 0.49541 |
|  | p | | 0.7205 | 0,7621 | 0.0138 |
|  | n | | 41 | 24 | 24 |
| **sCD163** | r | | -0.33859 | -0,27826 | -0.11151 |
|  | p | | 0.0283 | 0,188 | 0.5956 |
|  | n | | 42 | 24 | 25 |
| **Fractalkine/CX3CL1** | r | | 0.01141 | -0,08132 | 0.24558 |
|  | p | | 0.9428 | 0,7056 | 0.2367 |
|  | n | | 42 | 24 | 25 |
| **I-TAC/CXCL11** | r | | 0.08988 | 0,05614 | -0.18314 |
|  | p | | 0.5714 | 0,7945 | 0.3809 |
|  | n | | 42 | 24 | 25 |

**Table S5: Impact of corticosteroids and biotherapies use on concentrations of the significant biomarkers**

Comparison of concentrations of BDNF, I-TAC/CCL11, sCD163 and Fractalkine/CX3CL1, the biomarkers which were relevant to discriminate pSS from RA or SLE, depending on treatment by corticosteroids or biotherapies.

Abbreviations: n: number of patients included in the analysis; WMW: Wilcoxon-Mann-Whitney test; p: p-value.

|  |  |  | **Corticosteroids** | | | | | **Biotherapy** | | | |
| --- | --- | --- | --- | --- | --- | --- | --- | --- | --- | --- | --- |
| **Biomarker** | **Parameters** | **Total population** | **No corticosteroid** | **Corticosteroids** | | **Test** | **p** | **No biotherapy** | **Biotherapy** | **Test** | **p** |
| BDNF  (pg/ml) | n | 93 | 74 | 19 | WMW | | 0.27 | 72 | 21 | STUDENT | 0.05 |
|  | Mean  (± SD) | 18342.02  (± 9461.33) | 18807.15  (± 9071.03) | 16530.47  (± 10930.30) |  |  |  | 17295.92  (± 9255.25) | 21928.67  (± 9499.42) |  |  |
|  | Median  (Q1; Q3) | 19679.0  (13139.0;23825.0) | 20311.0  (13481.0;23825.0) | 15905.0  (6955.0;25025.0) |  |  |  | 17616.0  (10968.5;23163.0) | 21903.0  (18698.0;26535.0) |  |  |
|  | Median  (min; max) | 19679.0  (1206.0;41394.0) | 20311.0  (1206.0;41394.0) | 15905.0  (2378.0;40083.0) |  |  |  | 17616.0  (1206.0;40503.0) | 21903.0  (2157.0;41394.0) |  |  |
| I-TAC/  CXCL11  (pg/ml) | n | 95 | 76 | 19 | WMW | | 0.91 | 74 | 21 | WMW | 0.13 |
|  | Mean  (± SD) | 54.28  (± 135.54) | 40.92  (± 48.57) | 107.74  (± 286.96) |  |  |  | 61.99  (± 152.70) | 27.14  (± 15.67) |  |  |
|  | Median  (Q1; Q3) | 28.0  (17.0;46.0) | 30.0  (17.0;46.0) | 25.0  (18.0;53.0) |  |  |  | 30.0  (19.0;49.0) | 21.0  (17.0;42.0) |  |  |
|  | Median  (min; max) | 28.0  (5.0;1275.0) | 30.0  (5.0;383.0) | 25.0  (10.0;1275.0) |  |  |  | 30.0  (5.0;1275.0) | 21.0  (8.0;55.0) |  |  |
| sCD163  (pg/ml) | n | 95 | 76 | 19 | WMW | | < 0.01 | 74 | 21 | WMW | 0.90 |
|  | Mean  (± SD) | 86383.61  (± 35192.43) | 90440.68  (± 34940.11) | 70155.32  (± 32149.05) |  |  |  | 86266.59  (± 34594.86) | 86795.95  (± 38110.62) |  |  |
|  | Median  (Q1; Q3) | 78569.0  (64491.0;103024.0) | 87085.5  (70645.5;107583.0) | 65115.0  (48192.0;75579.0) |  |  |  | 79557.5  (65115.0;102747.0) | 76140.0  (62890.0;112124.0) |  |  |
|  | Median  (min; max) | 78569.0  (27626.0;193069.0) | 87085.5  (27626.0 ; 193069.0) | 65115.0  (30332.0;180278.0) |  |  |  | 79557.5  (29047.0;193069.0) | 76140.0  (27626.0;180408.0) |  |  |
| Fractalkine/  CX3CL1  (pg/ml) | n | 95 | 76 | 19 | WMW | | 0.58 | 74 | 21 | WMW | 0.36 |
|  | Mean  (± SD) | 335.77  (± 175.03) | 327.71  (± 163.69) | 368.00  (± 216.72) |  |  |  | 347.18  (± 191.73) | 295.57  (± 87.34) |  |  |
|  | Median  (Q1; Q3) | 292.0  (239.0;392.0) | 281.0  (241.5;388.5) | 327.0  (231.0;428.0) |  |  |  | 310.5  (231.0;405.0) | 272.0  (249.0;332.0) |  |  |
|  | Median  (min; max) | 292.00  (115.0;1233.00) | 281.0  (115.0;1233.0) | 327.0  (131.0;1059.0) |  |  |  | 310.5  (115.0;1233.0) | 272.0  (206.0;607.0) |  |  |
